# Transactions of the Buffalo Obstetrical Society

**Published:** 1886-01

**Authors:** 


### Society Reports.

### Transactions of the Buffalo Obstetrical Society.

*Stated Meeting, September 22, 1885.*

The President, Dr. W. W. Potter, in the chair; Dr. W. H. Thornton, Secretary.

Dr. M. Hartwig reported a case of

### DOUBLE PRESENTATION, the head and one foot appearing simultaneously at the brim. He was summoned by telegraph to aid another practitioner, and upon examination he was greatly puzzled to find a foot presenting at the same time with the head. The foot was peculiarly loose in its joint. He came to the conclusion that the physician, in attempting to turn, had pulled the right foot over the shoulder, and nearly out of its socket. He passed his hand into the uterus, performed version, and extracted the child with comparative ease. The woman, he learned, died twelve days later from puerperal septicæmia. The physician in charge refused to wash out the uterus in accordance with his advice.

Dr. Hartwig also reported the following case, the only death in obstetrics he ever had. The woman had been already three days in labor when he was called. He found a cross-presentation, with the uterus well contracted. He succeeded in turning with comparative ease, though found some difficulty in introducing the hand, the os being contracted and resisting his efforts. After turning, considerable blood came, and, upon delivery of the child, he became satisfied that it would not stop until he removed the placenta. He found it impossible to bring it away entire, and he was compelled to tear it off in pieces. He made an antiseptic injection, but the woman was already sinking; he, therefore, prepared an arterial injection of salt and water, and made a few injections, but the woman soon died. He raised the question whether he could have saved the woman, if he had left the placenta to be detached by itself. He feared the uterus itself might have been torn, as the blood came so freely after extraction of the child.

Dr. Thomas Lothrop endorsed the action of Dr. Hartwig in removing as much of the placenta as possible. He also stated that when women had borne a large number of children, they were quite apt to have complications in the later pregnancies. He related a case of breech presentation, complicated with

A LARGE SCROTUM.

He had attended the woman eight or nine times previously, all having been normal labors. This time he found some difficulty in differentiating the presentation. The examining finger first met quite a bag, though the bag of waters had previously broken; going a little higher it met on one side a hard bone, and, passing to the other side, another one. Soon it passed into what proved to be the anus, but there was no meconium. He finally decided that the bag was the scrotum, and very soon the penis came down, confirming that opinion. He mentioned the case as an example of some of the puzzling little features of obstetric cases.

Dr. R. L. Banta gave the history of a case of puerperal septicæmia, in which the treatment was antipyrin and antiseptic washes (intra-uterine) of corrosive sublimate. One night the temperature went up to 104° F. The patient was given fifteen grains of antipyrin every hour, until the temperature fell below 100° F. Eight doses were administered before this was accomplished, from which time she went on to good recovery.

Dr. P. W. Van Peyma called attention to the fact that it was generally taught in the schools that the first stage of labor was free from danger. His experience led him to disagree with that teaching. In one of his cases the cervix was indurated, and he was obliged to use Barnes' dilators before progress was obtained. In another, the woman was in labor nearly a week before the os was dilated, when he was finally called. In this case, the child was still-born, though it was alive until within an hour of its birth; the second stage was not more than an hour in duration. The contraction was very violent, and he gave anodynes to modify the pain, and applied belladonna locally. In a recent case, the second stage did not last more than an hour; there was no delay in the delivery of the head; yet the child was born dead, and he was at a loss to account for it. The waters had discharged about forty-eight hours before the delivery of the child, and, he judged, the pressure had occurred before the second stage. He believed it would have been better to have dilated the os, and thus hurried on the first stage, than to have followed the old rule.

Dr. W. D. Greene related the history of a case of puerperal convulsions which died undelivered. The woman was in the beginning of the eighth month of pregnancy, had seventeen convulsions, and there was no dilatation of the os. The treatment was morphia, by hypodermic injection, ¼ to ⅓ grain every hour, until it affected the pupils. Dilatation was attempted, but failed. The woman lived about ten hours after his first visit; her urine was loaded with albumen.

Dr. Hartwig thought the first thing to do in puerperal convulsions was to induce profuse perspiration, with a view to relieve the kidneys, the same as in any other form of parenchymatous nephritis.

---

*Stated Meeting, October 27, 1885.*

The President, Dr. W. W. Potter, in the chair.

Dr. Wm. B. Hawkins read a paper, entiled,

OBSERVATIONS ON DYSMENORRHŒA.

He stated that perfect general health and good physical development were necessary to the performance of the menstrual function without pain. Instead of this ideal condition, we most often had the opposite presented, in the person of a girl whose mental development had been forced at the expense of her physical, who prematurely arrived at the period of puberty with imperfectly-developed sexual organs, and a hypersensitive nervous system—one who possesses a predisposition to early menstrual development. He believed the prime factors in the production of this predisposition were, first, the faulty relation, or a misconception of the proper relation which children and young girls bear to society; and, second, a faulty educational and labor system, both in theory and practice. He regarded it the exception, rather than the rule, to allow and encourage children of both sexes, but more especially girls, to pass through the period of childhood as nature intended. They were generally put on an open-all-day-family-pride exhibition, the child starting out in life as a wonderful, educated baby, thus interrupting good, physical development, and deteriorating resisting force. The difference was not greater between such a child and one of robust physique, than between a hot-house plant and one that had borne the storm, as well as the warmth and sunshine, of a season.

Later, the girl was apt to diverge still farther from what should be a natural one; the continued excitement of her artificial environment, together with her educational tasks, soon developed the nervous system at the expense of physical growth, and to the deterioration of her already enfeebled constitution. When the period of puberty was reached, the other factor, *a total disregard of sex in education*, became active. The second, when added to the first, would almost certainly produce derangement of the menstrual function. The same faulty principle was in operation in business and the trades, the girl in either case being obliged to do her work in a boy's or man's way, and was constantly encouraged to put down and disregard her sex and nature's demands. He believed these considerations constituted the key-note to the nature and etiology of dysmenorrhœa; in other words, it was due, as a rule, to constitutional causes, and, therefore, deserved to be classified as a distinct disease.

He next referred to the different views held by authors on the subject, many of whom gave prominence to the theory that dysmenorrhœa was but a system of displacement or deformity of the uterus. Grailly Hewett practically accepted but one theory, the obstructive, and believed stenosis or obstruction, due to flexions of the uterus, to be the most common cause of the disease. This very plausible theory had won general acceptance among physicians, particularly on account of the good results from mechanical treatment; but he thought those results could be as readily comprehended upon the theory of nervous influence. He related two cases in illustration of this point. Both complained of, and sought relief for, sterility; and each presented very marked anteflexion, with elongated cervix, but only one suffered pain at the menstrual period. He practiced forcible dilatation in both, and effected a cure, for the present at least, of the dysmenorrhœa in the patient who suffered with it. Of these two patients, the one suffering no dysmenorrhœa presented the more marked anteflexion, and greater *apparent* obstruction at the internal os. Dilatation probably relieved the dysmenorrhœa by changing the existing disturbed condition of the sentient nerves, in the same manner as a local neuralgia is relieved by stretching a nerve, or as stretching the anus relieves the pain of, and cures, an anal fissure.

He did not claim that there were no cases of obstructive (mechanical) dysmenorrhœa, but it did not follow that the case was one of that sort, merely because it was relieved by dilatation, and quoted Emmet and J. E. Burton in support of this view. It was in pure and uncomplicated cases that the true nature of a disease was best determined. In such cases of dysmenorrhœa, it was probable that the sentient nerves of the endometrium were in a state of hyperæsthesia. This condition was seen reflected in the highly sensitive and impressionable temperament, and was generally accompanied by faulty development of the sexual and generative organs, as well as a deteriorated condition of general health, a penalty too often of a poor inheritance, a forced education, an emotional life, and a bad hygiene. In such a case, menstrual pain would be excited by the congestion consequent upon the periodical flushings of the poorly developed tense, and resisting tissue with blood. It was not difficult to explain the nature of the disease upon a purely constitutional and neurotic basis, when so many cases were relieved, and even permanently cured, by constitutional and hygienic measure alone. Mathews Duncan, Emmet and Mundé were referred to as supporters of the constitutional, neuralgic and hystero-neurotic theory of the disease; and Dr. C. D. Palmer's paper on "Dysmenorrhœa," published in the transactions of the American Gynæcological Society for 1883, was also quoted from in substantiation of the same view.

He next called attention to that form of the disease depending upon gouty and rheumatic diathesis, the study of which had been too much neglected, he judged, by the paucity of literature on the subject. A very characteristic symptom of dysmenorrhœa, dependant upon the rheumatic diathesis, was severe vertical headache, which was apt to persist during the inter-menstrual period. The recognition of this fact in a recent case had enabled him to successfully treat it, after it resisted the ordinary methods.

In considering the treatment of dysmenorrhœa, he called attention to the necessity of instructing parents, teachers and others in regard to the part they ought to take in preventing the acquirement of a predisposition to the disease. He regarded the failure to recognize sex, in education and business, as a prime etiological factor in the malady, and urged the importance of instructing the laity in the laws of prevention. Girls should be excused from special mental effort during the menstrual periods, and at all times great care should be exercised in prohibiting *anything* from the interference with continued growth and perfect physical development. In young and married women, treatment should be purely constitutional for a time, with a view to correct faults in the nervous system, and considerable attention should be paid to personal hygiene. Iron and arsenic were the two most important drugs, singly or combined, with which to aid the general management of these cases, and the former should be given in large doses, and continued for a long time. The systematic use of Turkish and Russian baths, and massage, he had found excellent adjuvants; and in cases complicated with gouty or rheumatic diathesis, remedies appropriate to those conditions should be adopted.

In cases where no improvement followed this plan, local examination, and adoption of local expedients, were imperatively demanded. Dilatation offered, in such cases, a strong probability of relief, and it could be accomplished by either three methods: First, gradually, by means of graduated bougies or sounds; second, forcibly, by any of the strong two-bladed dilators; or third, by an intermediate process, as by the use of Molesworth's four-bladed instrument.

The continued good results which are often obtained by the repeated introduction of bougies, which produce very little dilatation, tended to prove that the condition was rather one of neuralgia than of obstruction. For the treatment of the immediate attack, he advocated the use of permanganate of potash. Beginning its use three or four days before the expected flow, and continuing it until the flow was well established, he had seen the pain mitigated, and often disappear altogether, after two or three periods. Bartholow had attributed its benefits to the power which the drug has of giving up oxygen and ozone to the system.

DISCUSSION.

Dr. P. W. Van Peyma said that the practice of attributing any one symptom always to one condition, in an etiological sense, was contrary to the usual law of disease; therefore, he warned against falling into the error of always ascribing dysmenorrhœa, as well as many other disorders of women, to faulty methods of education. He believed that the general methods and habits of society and fashion, to which so many young women yield such unswerving devotion, were quite as much at fault in this matter; and that late hours, attendance upon balls, parties, and other society gatherings, when the menstrual molunen was on, were often fully as deleterious as overstudy.

Dr. C. C. Frederick referred to the differences of opinion as to the causes and treatment of dysmenorrhœa among prominent members of the profession, whose opportunities for observation were, perhaps, equally great on both sides. Taken all in all, he had obtained the best results in his practice from the method recommended by Goodell, viz.: Forcible dilatation under ether.

Dr. J. W. Keene had benefited some cases by rapid dilatation; had not, on the whole, succeeded well with medication alone; but found it necessary, oftentimes, to continue the dilatation indefinitely. He believed the cause to be generally neurotic, and questioned the causal relations of so-called obstructions to displacements.

Dr. R. L. Banta agreed with the essayist in the main, and, where indicated, had obtained excellent results from the employment of Blaud's pill in nine-grain doses—about three times the usual amount. He had found Molesworth's four-bladed expanding dilator a satisfactory instrument for rapid dilatation. Related a case that came under his notice when dilatation had previously been performed with sponge tents, resulting in cellulitis, which had bound down the uterus.

DR. M. HARTWIG commented upon the general excellence of the paper, and was particularly pleased with the writer's advocation of general treatment. Dysmenorrhœic pain usually preceded the flow; therefore, in such cases, pain could not be due to obstructive flexions, or to impossibility of the escape of blood. Cause might sometimes be due to previous inflammations, which had bound down the uterus. Anteflexion could not produce much mechanical obstruction to the escape of blood; but retroflexion was more likely to do so. He had met with very few cases of dymenorrhœa in the country, but had observed them more frequently in the city, and thought heredity played an important etiological part.

DR. G. E. FELL considered the effect of school-life upon young women an important question in connection with this subject. The social influences were apt to come on later in life, and, therefore, do not present the same causal inferences. The time to exact obedience to strict physical and mental hygiene was during the school-girl period of life.

The President would group the causes under three heads: First, the various forms alluded to in the paper as constitutional varieties, and those in which heredity was a factor; second, the obstructive group, embracing all cases where there were faulty channels of exit, whether from flexions, stenosis, or growths; and, third, the neurotic varieties of the disease. It was curious to note the almost paradoxical relations of flexions to dysmenorrhœa. Not all flexions caused, or were even associated with, dysmenorrhœa; and dysmenorrhœa co-existed sometimes with flexions in which there were free channels of exit, or at least no obstructions by reason thereof.

In such cases, the disease was due, no doubt, to tissue changes resultant from the flexions, and not, as had been erroneously supposed by many, to mere mechanical infringement upon the lumen of the cervical canal. These cases were, nevertheless, benefited by dilatation through the overstretching of the neurotic tissue near the internal os. These were the cases in which good generally resulted from repeated gradual dilatation with bougies. Rapid dilatation under ether was also good practice in certain selected cases of dysmenorrhœa, where other measures had failed. He believed, with the author of the paper, that faulty education, during early school years, was responsible for much of this kind of trouble in young women.

